# “FIND Technology”: investigating the feasibility, efficacy and safety of controller-free interactive digital rehabilitation technology in an inpatient stroke population: study protocol for a randomized controlled trial

**DOI:** 10.1186/s13063-016-1318-0

**Published:** 2016-04-16

**Authors:** M. L. Bird, J. Cannell, M. L. Callisaya, E. Moles, A. Rathjen, K. Lane, A. Tyson, S. Smith

**Affiliations:** Faculty of Health, University of Tasmania, Locked Bag 1322, Launceston, 7250 Australia; Tasmanian Health Organisation, Launceston, Australia; Menzies Medical Research Institute, Medical Science Precinct 17 Liverpool Street, Hobart Tasmania, 7000 Australia; University of the Sunshine Coast, 90 Sippy Downs Dr, Sippy Downs Queensland, 4556 Australia

**Keywords:** Rehabilitation, Exergames, Exercise, Activity

## Abstract

**Background:**

Stroke results in significant disability, which can be reduced by physical rehabilitation. High levels of repetition and activity are required in rehabilitation, but patients are typically sedentary. Using clinically relevant and fun computer games may be one way to achieve increased activity in rehabilitation.

**Methods/design:**

A single-blind randomized controlled trial will be conducted to evaluate the feasibility, efficacy and safety of novel stroke-specific rehabilitation software. This software uses controller-free client interaction and inertial motion sensors. Elements of feasibility include recruitment into the trial, ongoing participation (adherence and dropout), perceived benefit, enjoyment and ease of use of the games. Efficacy will be determined by measuring activity and using upper-limb tasks as well as measures of balance and mobility. The hypothesis that the intervention group will have increased levels of physical activity within rehabilitation and improved physical outcomes compared with the control group will be tested.

**Discussion:**

Results from this study will provide a basis for discussion of feasibility of this interactive video technological solution in an inpatient situation. Differences in activity levels between groups will be the primary measure of efficacy. It will also provide data on measures of upper-limb function, balance and mobility.

**Trial registration:**

ACTRN12614000427673. Prospectively registered 17 April 2014.

## Background

In the US alone, one person per minute has a stroke, and although death rates have declined over the last decade, the burden of disease remains high [13]. Physical rehabilitation has the potential to positively impact functional outcomes and improve this burden; however, this requires a high dose of therapy. A significant factor limiting rehabilitation outcomes is low levels of patient activity [10]. Observational studies in different countries have found that patients after stroke in rehabilitation are surprisingly inactive for the vast majority of the waking day. For example, only 13 % of a stroke unit patient’s day is typically spent in activities related to functional outcome, such as active therapy or walking practice [2]. Many rehabilitation activities, aimed at stimulating neuroplasticity, are by their very nature repetitive and tend to be tedious [19]. One method by which engagement with rehabilitation programs and levels of activity could be improved involves the use of fun and engaging video games.

Commercial, off-the-shelf devices such as the Microsoft Xbox Kinect (Microsoft Corporation, Redmond, WA, USA) are relatively inexpensive and use motion capture and feedback technologies with potential for use in rehabilitation. Interactive video games increase adherence to and enjoyment of exercise in the general population [1] and have the potential to increase the dose of repetitive exercise completed by people with reduced mobility. Exercise-based video games could be used to increase exercise dose during therapy and to enable exercise outside of therapy hours. This is true both in inpatient and outpatient rehabilitation settings as well as at home after discharge from hospital.

In particular, the Kinect for Xbox 360, or simply Kinect, is a “controller-free gaming and entertainment experience” by Microsoft for the Xbox 360 video-game platform and is now also supported by PCs via Windows 8. It enables users to control and interact with the Xbox 360 without the need to touch a game controller, through a user interface using gestures and spoken commands. Kinect enables full-body depth-based three-dimensional motion-capture, facial recognition and voice recognition capabilities. This differentiates it from previous generations of interactive technologies that have been used in rehabilitation.

Despite the promise of such low-cost, consumer-based technologies, many, if not all, off-the-shelf video-game solutions are inappropriate for individuals with functional impairment [16]. There is an opportunity for purpose-built, clinically relevant video game-based rehabilitation to add significant value to current rehabilitation practice. Jintronix, a Montreal-based company, has recently launched a Kinect-based rehabilitation system, Jintronix Rehabilitation System (JRS), which provides an easy-to-use software solution (JRS WAVE) for patients to use. The software solution has been designed in collaboration with physical and occupational therapists and draws upon the motor relearning recommendations by Carr and Shepard [5]. As such, upper limb, sitting balance, standing balance and stepping rehabilitation tasks have been programmed in the JRS WAVE as fun and engaging video games that can be played at a number of different levels of complexity and speed. The system is also capable of automatically measuring changes in the range, speed and quality of motion to give patients instant feedback on their progress.

A second feature of the JRS WAVE is a cloud-based client management telehealth system for clinicians to recommend rehabilitation tasks and track and record performance of those tasks (JRS PORTAL). The PORTAL allows clinicians to provide patients regular updates and information on what has happened to them with daily, weekly or monthly progress reports on their rehabilitation, either face-to-face or remotely.

The proposed project will evaluate the feasibility, efficacy and safety of the JRS WAVE for use in an Australian stroke inpatient rehabilitation context. Elements of feasibility include recruitment into the trial, ongoing participation (adherence and dropout), perceived benefit, and enjoyment and ease of use of the games. Efficacy will be determined by measuring physical activity (using an accelerometer) and using upper-limb tasks as well as measuring changes in balance and mobility over time between the two groups. Adverse events will be monitored and changes in pain and fatigue with the interventions will be used to determine safety of the system. We will test the hypothesis that the intervention group will have increased levels of physical activity within rehabilitation and improved physical outcomes compared with the control group.

## Methods/Design

A single-blind randomized controlled trial will be conducted on two rehabilitation wards of a secondary referral hospital. Participants will be randomly assigned into the JRS WAVE intervention that will last for the duration of the stay on the ward, with a minimum dose of 8 days and maximum of 40 or usual care. All participants will provide written informed consent, and ethical clearance has been received.

### Participants

#### Inclusion

Eligible participants will be adults admitted to the two rehabilitation wards at the Launceston General Hospital with reduced mobility after haemorrhagic or infarct cerebrovascular accident of recent onset (less than 6 months) with a clinician-assessed capacity for improvement in mobility.

Exclusion criteria will be the following: a marked cognitive impairment so that instructions are not able to be carried out (determined by the therapist); insufficient English language skills to participate in rehabilitation and no available interpreter; a medical condition precluding exercise (unstable cardiac disease, uncontrolled hypertension, uncontrolled metabolic diseases, large abdominal aortic aneurysm or a weight-bearing restriction); or severe receptive or expressive dysphasia of severe visual impairment.

#### Sample size and justification

Sample size calculation was based on previously reported clinical trial data and based on the outcome measure of functional reach, that provided baseline mean (standard deviation) data of 25.6 (7.4) cm [14]. Researchers determined that a clinically relevant difference of 3.7 cm would require a sample size of 63 (*P* <0.05, power 80 %). Seventy-four participants will be recruited to allow for a 15 % dropout rate. The proposed size of this study is comparable to more recently published trial data, which suggest that a clinical difference of 3 cm would require 78 participants [3].

#### Recruitment

All patients admitted to the two rehabilitation wards during the trial period will be screened for inclusion by a senior neurological physiotherapist. Eligible patients will be given information about the project and if they are interested, a research assistant (physiotherapist) will be contacted to provide further information. Written consent will be obtained before participation in the project. Demographic and background information will be collected. Ethical approval for this study has been received from the Human Research Ethics Committee (Tasmania) Network (approval number H0013769).

#### Study design

All participants will undergo two measurement sessions by a physiotherapist blinded to group allocation: one on entry into the study (pre-test) and one after the 8-week period (post-test) or prior to discharge if discharged before 8 weeks. All pre-test measures will be completed prior to randomization.

#### Randomization

Allocation into intervention or control group will be performed by using a computer-generated random number schedule with variable block sizes 2–6. Generation of the randomization sequence will be generated by a researcher not involved in recruitment or assessment. Group allocation will be concealed by using consecutively numbered opaque sealed envelopes, opened in the presence of the participant after completion of assessment.

### Intervention and usual care

Participants in both groups will be assessed by a physiotherapist who will deliver individualised targeted therapy to meet their specific needs.

#### Usual care

In addition to receiving individualised therapy, the usual care group will have a therapist prescribe a series of repetitive exercises (e.g., practice of arm activities, standing up or stepping) in a group setting with physiotherapists or physiotherapy assistants. Usual care varies between the two wards used in this trial. Specific information about usual care for each ward and the delivery plan is provided in Appendix 1.

#### Intervention group

JRS WAVE technology use will replace group exercise prescribed in the usual care group or allied health assistant sessions which are usual care on ward two. Participants randomly assigned to the intervention group of this trial will receive individualised prescription of appropriate technology-based exercises to enhance physical activity and mobility. In addition to receiving individualised targeted therapy, each participant will be asked to use the intervention technologies for up to 1 hour each day. As such, both groups will be matched for time in rehabilitation to determine the impact of the technology-based intervention compared with group exercise.

Therapy staff will teach participants to use the equipment and develop individualised exercise programs to enable participants to use the equipment safely. To minimise the risk of contamination, use of the technologies will be done out of sight of control group participants where possible.

#### Quality control and feasibility

Feasibility will be monitored by recording numbers of potential participants who are recruited into the study, the number of sessions undertaken out of the possible number of sessions available during the inpatient stay, and reasons for missed sessions.

Assessment of post-study outcome measures will be performed by a neurological physiotherapist who is not involved in the provision of therapy to that client. This will ensure blinding of assessment and maintain the quality of this project. It is not possible to blind the participant as to the group allocation.

#### Efficacy

##### Physical activity

All participants will have their activity monitored for 1 week during the trial by using an accelerometer (ActivPal; PAL Technologies, Glasgow, UK). These devices have been validated in older people with impaired function [18]. Data will be analysed in each group to determine the amount of time spent in activities of low, moderate and high energy expenditure. Energy expenditure during rehabilitation will also be compared between groups.

##### Upper-limb function

Upper-limb function will be measured by using the Upper Arm Function component of the Modified Motor Assessment Scale. This test uses a six-point scale to measure a progression of proximal arm strength and has been shown to be reliable in this population [12]. The box and block test will also be used to provide quantitative results [8].

##### Sitting balance

Sitting balance will be measured by using the standardised sitting balance test, which uses a four-point rating scale to measure sitting balance from poor to normal. It correlates significantly with other measures of function [17].

##### Standing balance

Standing balance in the anterior-posterior direction will be measured by using the Functional Reach test [9], and balance control in the medio-lateral by using the Lateral Reach test [4]. Functional Reach measures dynamic balance control during a self-generated perturbation anteriorly. Participants will stand next to a wall and use their less-affected arm to measure reach distance. Lateral reach measures side-to-side reach ability and will be measured with the participant in an upright position and reaching to the side. The shoulder landmark will be used for each side to ensure that shoulder range does not limit the test. This test can be performed in a seated position if the therapist deems this necessary for safety.

##### Dynamic balance

Dynamic balance will be assessed by using the step test, which involves tapping the foot on and off a step within a 15-second time frame and recording the number of repetitions [11].

##### Mobility

Mobility will be measured by using a 10-meter walk test. Participants will be asked to walk 10 meters using their usual gait aid, and measurements of time taken, stride length, cadence and rate will be recorded [7]. Functional mobility will also be recorded by using the “timed up and go” test [15].

#### Safety

Adverse events during both intervention care and usual care will be recorded. Also, after each session, participants will provide the following data of their experience participating in the group or using the technology:

- Borg rating of perceived exertion scale
- Visual Analogue Scale (VAS) (pain)
- VAS (fatigue)
- 5-point Likert Scale (enjoyment)
- 5-point Likert Scale (perceived benefit).

Therapists involved in the delivery of the JRS WAVE will be invited to complete an anonymous survey that asks for their feedback on the utility of the system. Perspectives on potential benefits and harm will also be recorded.

### Data Collection

**Fig. 1:**
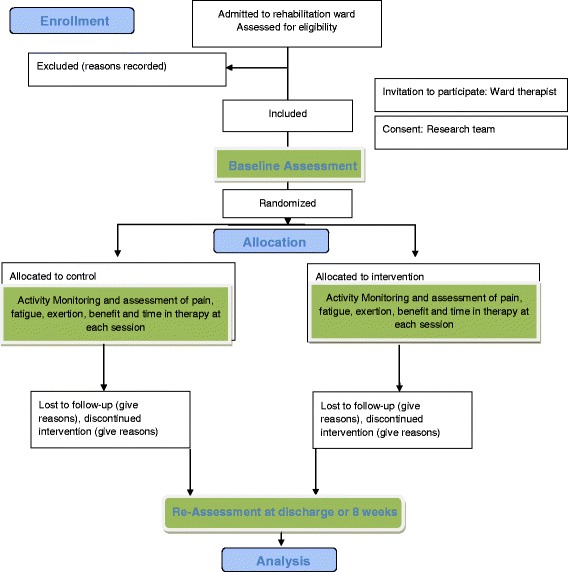
Feasibility Interactive Digital (FIND) technology protocol flow diagram

Flow through the study is identified in Fig. 1.

### Data analysis

Data collected during the trial will allow us to describe the participants’ changes in upper-limb function, balance and mobility by using *t* tests or Mann–Whitney *U* tests to compare normally and non-normally distributed data, respectively. Repeated measures analysis of variance will be used to compare changes in the outcome measures over time and between groups (intervention versus control). The level of significance will be set at 0.05.

## Discussion

The proposed project will discuss the feasibility and efficacy of the JRS WAVE. Uptake from screening to recruitment, adherence to the intervention and rates of dropout will be discussed. Feedback from clinicians in terms of system utility will be explored. Feedback from clients on the perceived benefits of this type of rehabilitation and enjoyment and ease of use of the games will also be discussed. Changes in pain and fatigue within each session and between the intervention and control groups will be analysed. Differences in activity levels during the two different interventions (JRS WAVE versus group exercise) will be investigated. Between-group differences for outcome measures of upper-limb function as well as balance and mobility will be investigated.

The primary outcome of efficacy consists of the differences in activity levels from using video technology in rehabilitation compared with usual rehabilitation. It will also provide data for new controller- and device-free motion capture software on measures of upper-limb function, balance and mobility. Much of the published research reviewing the effectiveness of interactive technology games with commercial devices has not used motion capture software in a controller-free situation. For example, the wii™ system for balance requires standing on a platform and the upper-limb tasks require the client to hold (or have strapped to their hand if they do not have grasp capacity) a device that the sensor recognises [6].

This software has been iteratively designed with feedback from the clinicians to form a system that meets the needs of stroke clients in the acute hospital setting as they begin their rehabilitation pathway. This system is vastly configurable, giving the clinicians the ability to tailor the program and modify it in an ongoing way to meet the needs of the clients. The different levels of disability of the clients enrolled will provide challenges when determining between-group differences.

Strengths of this study include that it collects feasibility data from both clients and clinicians. It is a well-powered study with an active control group to allow comparisons across a range of functional measures. Also, both groups are matched for time in rehabilitation (rather than the intervention as additional therapy) and this will allow direct comparison of the groups.

The novel nature of this bespoke software will provide valuable information to clinicians and researchers looking to improve engagement with rehabilitation, activity within rehabilitation and improved client outcomes in a cost-effective way.

### Trial status

Recruitment for this study is still under way.

## Appendix

**Table 1:** FIND Study Therapy Delivery Plan

	Control	Intervention
Ward One easily fatigued client	Daily individual therapy Functional based	Daily individual therapy Alternating daily functional based therapy and Jintronix
Ward One standard client	AM Group Therapy	AM Jintronix
	PM Individual as required	PM Individual as required
Ward Two easily fatigued client	AM short AHA session	1 short AHA session
	PM short AHA session	1 short Jintronix
	Weekly PT review	Weekly PT review
Ward Two standard client	Daily AHA session	Daily Jintronix + gait/stairs/bed TF
	1-2x / week PT session	1-2x / week PT session
Ward Two client with higher tolerance	Daily AHA session	Daily Jintronix + gait/stairs/bed TF
	4 days / week seated ex group	4 days / week seated ex group
	1-2x / week PT session	1-2x / week PT session
Ward Two client with high tolerance	Daily AHA session	Daily Jintronix + gait/stairs/bed TF
	4 days / wk functional ex group	4 days / wk functional ex group
	1-2x / week PT session	1-2x / week PT session

PT Physiotherapy, AHA Allied Health Assistant, TF transfers

## Abbreviations

JRS: Jintronix Rehabilitation System
VAS: Visual Analogue Scale
