# Erratum to: ‘“FIND Technology”: investigating the feasibility, efficacy and safety of controller-free interactive digital rehabilitation technology in an inpatient stroke population: study protocol for a randomized controlled trial’

**DOI:** 10.1186/s13063-016-1376-3

**Published:** 2016-05-19

**Authors:** M. L. Bird, J. Cannell, M. L. Callisaya, E. Moles, A. Rathjen, K. Lane, A. Tyson, S. Smith

**Affiliations:** Faculty of Health, University of Tasmania, Launceston, Australia; Tasmanian Health Organisation, Launceston, Australia; Menzies Medical Research Institute, Launceston, Australia; University of the Sunshine Coast, Sippy Downs, Australia

Unfortunately, the original version of this article [1] contained an error. An acknowledgement was included incorrectly.

Support for this trial was received from the National Stroke Association (Australia). We have also recieved funding to assit with publication costs from The Tasmanian Health Organisation (North).

The correct acknowledgement can be found below:

Support for this trial was received from the National Stroke Association (Australia). We have also received funding to assist with publication costs from The Tasmanian Health Service (Northern Region).
